# Properties of Banana (*Cavendish* spp.) Starch Film Incorporated with Banana Peel Extract and Its Application

**DOI:** 10.3390/molecules26051406

**Published:** 2021-03-05

**Authors:** Chanitda Taweechat, Tipapon Wongsooka, Saroat Rawdkuen

**Affiliations:** 1 Food Science and Technology Program, School of Agro-Industry, Mae Fah Luang University, Chiang Rai 57100, Thailand; 5731401014@lamduan.mfu.ac.th (C.T.); 5731401030@lamduan.mfu.ac.th (T.W.); 2 Unit of Innovative Food Packaging and Biomaterials, School of Agro-Industry, Mae Fah Luang University, Chiang Rai 57100, Thailand

**Keywords:** active packaging, banana starch, film, peel extract, minced pork

## Abstract

The objective of this study was to develop an active banana starch film (BSF) incorporated with banana peel extract. We compared the film’s properties with commercial wrap film (polyvinyl chloride; PVC). Moreover, a comparison of the quality of minced pork wrapped during refrigerated storage (7 days at ±4 °C) was also performed. The BSF with different concentrations of banana peel extract (0, 1, 3, and 5 (%, *w/v*)) showed low mechanical properties (tensile strength (TS): 4.43–31.20 MPa and elongation at break (EAB): 9.66–15.63%) and water vapor permeability (3.74–11.0 × 10^−10^ g mm/sm^2^ Pa). The BSF showed low film solubility (26–41%), but excellent barrier properties to UV light. The BSF had a thickness range of 0.030–0.047 mm, and color attributes were: L* = 49.6–51.1, a* = 0.21–0.43, b* = 1.26–1.49. The BSF incorporated with banana peel extracts 5 (%, *w/v*) showed the highest radical scavenging activity (97.9%) and inhibitory activity of *E. coli* O157: H7. The BSF showed some properties comparable to the commercial PVC wrap film. Changes in qualities of minced pork were determined for 7 days during storage at ±4 °C. It was found that thiobarbituric acid reactive substances (TBARS) of the sample wrapped with the BSF decreased compared to that wrapped with the PVC. The successful inhibition of lipid oxidation in the minced pork was possible with the BSF. The BSF incorporated with banana peel extract could maintain the quality of minced pork in terms of oxidation retardation.

## 1. Introduction

Food packaging functions to reduce the rate of gas transfer between food and the environment and the control of oxygen and water vapor permeability allow the extension of the shelf life of foods [1]. The use of packaging from synthetic plastics causes serious environmental problems, giving rise to a demand for packaging alternatives from biodegradable materials. The disposal of synthetic packaging is difficult because it is non-degradable and non-recyclable, thus taking a long time to break down. Therefore, the use of biodegradable packaging materials can solve this problem to obtain environmentally friendly ones, which can be made from natural polymers such as proteins, lipids, and polysaccharides. Among polysaccharides, starch has received special attention; it is abundant, cheap, biodegradable, edible, and renewable [2,3,4]. Starch is an agricultural biopolymer found in a variety of plants including wheat, corn, rice, beans, potatoes, etc. However, a literature search via Scopus during the last sixteen years (2006–2021) found that the film made of banana starch already published only five research papers [2,5,6,7,8], and all of these mostly focused on characterization and the banana starch was not green *Cavendish* spp. The effectiveness and the applicability of the banana starch film on perishable food products were not found. 

Bananas are one of the major fruit crops in Thailand. It is a commonly consumed fruit and, worldwide, banana production reached a record of 114 million tonnes in 2017, up from around 67 million tonnes in 2000 (Food and Agriculture Organization: FAO, 2021). In Chiang Rai, about 4 tons per day of harvested green banana (*Cavendish* spp.) is wasted, and all rejected green bananas are normally disposed improperly. Therefore, preparing edible films from banana starch is an alternative for using this raw material to provide a benefit. The entire banana fruit is rich in bioactive compounds, such as phenolic constituents, carotenoids, vitamins, and dietary fiber [9,10]. Unripe banana naturally consists of more than 70% starch, with the remainder being protein, lipid, and fiber [11,12]. The polysaccharides in banana powder give extra hydrogen bonding contacts between polymer chains, which is responsible for the film-forming capacity [13]. In addition, banana starch can be used as a base material for preparing the biodegradable film with good gas barrier properties as well as nontoxic properties [8,14]. 

The incorporation of natural active agents into edible film can be used instead of chemical agents, which can improve the functional properties of film as well as maintain the quality of food. Banana peel has stronger antioxidant activity, greater phenolic compounds, and a higher mineral content than banana pulp. Additionally, banana peels were evaluated as having powerful antimicrobial activity against bacteria, fungi, and yeast [8,15,16,17]. Bioactive compounds such as flavonoids, tannins, phlobatannins, alkaloids, glycosides, and terpenoids are present in banana peel [15]. According to these reports, the incorporation of these into banana starch based film was not found, as well as the application of the developed film in the food system. 

Meat is one of the most perishable foods because of its nutritional composition. The spoilage of minced pork or other meat products is mainly caused by chemical and microbiological deterioration. The spoilage of fresh pork by microorganisms occurs mostly due to improper handling before and after it is slaughtered [18,19]. Lipid oxidation, protein degradation, and the loss of other valuable molecules are the consequences of the meat spoilage process. The objective of the present study was to develop and characterize unripe banana starch film incorporated with banana peel extract and apply the developed film to minced pork.

## 2. Results and Discussion

### 2.1. Properties of Banana Starch Film Incorporated with Banana Peel Extract

#### 2.1.1. Thickness

The thicknesses of banana starch film (BSF) incorporated with banana peel extract with different concentrations in comparison with polyvinyl chloride (PVC) film are presented in Table 1. Significant differences in thickness were observed between BSF and PVC film (*p* < 0.05). The BSF was four times thicker than the PVC film. PVC film had a thickness value of 0.010 mm, while the BSF had a thickness value range of 0.030–0.047 mm. This result also showed that when the concentrations of banana peel extract increased, the film thickness was also increased. However, the thickness of the BSF was not influenced by banana peel extract concentration (*p* > 0.05). According to Gutiérrez et al. [20], a greater interaction between the starch and plasticizer could result in thicker films probably due to the formation of hydrogen bonds between the glycerol and starch. Pérez et.al. [21] reported a significant increase in the thickness of cross-linked starch-based films derived from *Dioscorea trifida*. Cross-linking apparently strengthens internal bonds in starch, which increases molar volume [22]. This factor, together with a greater interaction between the starch and plasticizer during gelatinization, could result in thicker films. Film thickness generally affects the properties of films such as mechanical properties (tensile strength (TS) and elongation at break (EAB)), water vapor permeability, light transmission as well as film transparency.

#### 2.1.2. Mechanical Properties

For packaging films, good mechanical properties such as TS and EAB are required for the films to resist external stress and maintain their integrity, as well as to act as barriers during the packaging process. The mechanical properties of the BSF incorporated with banana peel extract at different concentrations in comparison with PVC film were expressed in terms of TS and EAB, as shown in Table 1. The BSF had a slightly decreased TS (43–31.20 MPa) and EAB (9.66–15.63%) when the banana peel extract was added, but no significant difference was observed in EAB (*p* > 0.05). The addition of glycerol to the film forming solution (FFS) significantly affected the TS of the BSF. As compared to PVC film, it showed a greater TS value (44.10 MPa) over the BSF. For the EAB, BSF still had a lower value than the PVC. According to Pelissari et.al. [6], this behavior could be explained by the differences in the amylose content of the films, since it is known that films rich in amylose exhibit good mechanical strength but little flexibility. High tensile strength is generally required, but deformation values must be adjusted according to the intended application of the films.

#### 2.1.3. Thermal Properties

The melting temperature (Tm) and enthalpy (ΔH) of the BSF incorporated with different concentrations of banana peel extract are shown in Table 1. The Tm and ΔH of the BSF incorporated with banana peel extract were in the range of 89.83–98.50 °C and 192.30–273.90 J/g, respectively. This hydothermic peak has been associated with the disruption of inter-chain interaction. According to Kaewprachu et.al. [23], the Tm of the films indicated the temperature and caused a disruption of the polymer interaction that formed during film preparation. Changes in Tm and ΔH values were observed in the control films (0%) and banana starch film incorporated with banana peel extract, regardless of the level of banana peel extract concentrations used (*p* < 0.05). Additionally, lower enthalpy values (ΔH) associated with the glass transition have been related to the weakening of the inter- and intra-molecular interactions between starch–starch chains [24]. The thermal properties of banana starch film were markedly affected by the concentration of banana peel extract used.

#### 2.1.4. Film Appearance and Color

**Figure 1:**
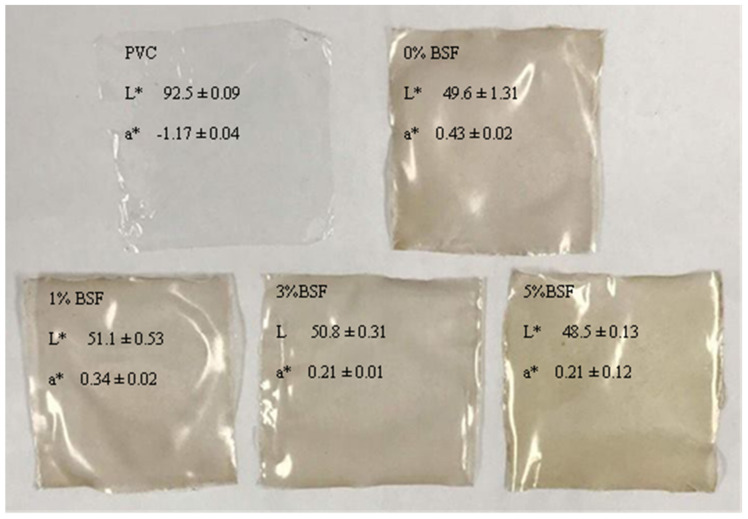
Appearance of banana starch films incorporated with banana peel extract with different concentrations.

The appearances of the BSF incorporated with banana peel extract at different concentrations in comparison with PVC film are shown in Figure 1**.** The lightness (L*), redness/greenness (a*) and yellowness/blueness (b*) values were significantly different when observed between the BSF and PVC film (*p* < 0.05). The results showed that the color attribute of the BSF had slightly increased L* and b* values when the banana peel extract content increased from 0 to 5 (%, *w/v*), but no significant difference was observed in b* value (*p* > 0.05). The a* value of the BSF was slightly decreased because of the browning color of the peel extract. According to the composition of banana peel, which consists of sugar and amino acid, powder preparation using an oven drying browning reaction may result in a dark brown color. When compared with PVC film, the BSF containing banana peel extract had slightly lower lightness, while redness and yellowness showed higher values than PVC film. These results are similar to those reported by Gutierrez et al. [20], which confirmed that films with higher amylose content are more opaque. According to these findings, it can be concluded that the addition of banana peel extract in the BSF might limit the use of the film only in packaging such as pouch bags for oil.

#### 2.1.5. Moisture Content, Film Solubility, Water Vapor Permeability

The moisture content of the BSF was affected by the incorporation of banana peel extract (*p* < 0.05) (Table 2). The moisture content of the experimental films gradually increased with increasing concentrations of banana peel extract. According to Verbeek and Bier [25], moisture content directly affects the banana starch films’ mechanical properties because of their characteristic hydrophilicity. Typically, films that contain larger amounts of water have high flexibility and low strength compared with others. Moreover, films with high moisture content, if used for application, can cause microbial growth on the surface of packaged food [19,23].

According to the visual observation, the BSF maintained its integrity after a 24 h dip in water. The solubility of the BSF was in the range of 26–41%, while the PVC film was not soluble in water (Table 2). Significant differences in film solubility were observed between the films incorporated with banana peel extract and the control film (*p* < 0.05). The film solubility of the BSF was increased with increasing concentrations of banana peel extract (*p* < 0.05). The highest amount of banana peel extract addition showed the highest film solubility (40.8%), while the lowest film solubility was found in the control film (26.3%). So, the soluble substances could escape from the film during immersion in distilled water. Banana peel extract could be released into distilled water due to its hydrophilic nature. In general, high film solubility may indicate poor water resistance [4]. Higher film solubility when compared with others may have the possibility of developing active food packaging, which can be easily soluble and can release active agents contained in the active film [17].

The water vapor permeability (WVP) of the BSF incorporated with banana peel extract at different concentrations in comparison with PVC film is shown in Table 2**.** The WVP of the BSF showed in the range of 3.74–11.0 × 10^−10^ g mm/sm^2^ Pa, while the WVP of the PVC film was 1.15 × 10^−10^ g mm/sm^2^ Pa. The WVP of the BSF was slightly increased when increasing banana peel extract (*p* < 0.05). The higher amount of moisture content in the banana peel extract incorporated films also led to higher WVP values. When compared with PVC film, the BSF had a lower WVP than that of the PVC film. Similar results have been reported by Ma et.al. [26], who reported that nanoparticles such as those in starch modified with citric acid also reduced the film WVP. Thus, the BSF could be related to film thickness (Table 1). A low WVP of the BSF may not suitable to apply as food packaging. However, the characteristics of the film depend on the specific requirements for food preservation. 

#### 2.1.6. Antioxidant Properties of the Films

The antioxidant activity of the BSF incorporated with banana peel extract at different concentrations is expressed in terms of DPPH radical scavenging activity, and the results are shown in Table 2. The BSF incorporated with 5 (%, *w/v*) banana peel extract showed the highest radical scavenging activity (97.9%). The addition of banana peel extract increased the radical scavenging activity of the film. The incorporation of banana peel extract 1 (%, *w/v*) into the film did not show any significant difference in the radical scavenging activity when compared with the control film (*p* > 0.05). This was mainly due to the effective phenolic compounds in banana powder and banana peel extract [8,27]. These results are similar to those reported by Ramirez-Hernandez et.al. [5], who showed DPPH radical scavenging activity results obtained for the active films. As resulted, the increase in the added amount of rosemary extract to the formulations led to edible films with higher polyphenol content. Moreover, the DPPH-radical scavenging activities of the films increased progressively when the polyphenol content in the samples increased. Therefore, BSF behavior could be useful for applications where a longer release time of the antioxidants is required.

#### 2.1.7. Light Transmission and Transparency

The transmission of UV (200–280 nm), visible light (350–800 nm) and the transparency of all the films are shown in Table 3. The light transmission in the UV ranges was 18.9–84.8%, while the transmission in the visible range was 31.7–91.9%. The transmission of UV and visible light decreased with increased concentrations of banana peel extract. This result suggests that banana peel extract treated film could prevent UV transmission and therefore could reduce food deterioration, especially the lipid oxidation that is induced by UV light. A lowered visibility of light transmission was observed when banana peel extract increased. According to these results, the light transmission of developed film depended on the amount of banana peel extract in the film. 

The transparency value of the resulting films was in the range of 2.85–3.23, while the transparency value of PVC film was 3.95 (Table 3). All films had no significant effects on the transparency in concentrations (3–5%, *w/v*)) of banana peel extract (*p* > 0.05). The transparency value of developed film decreased when increasing the banana peel extract concentration. According to Pelissari et al. [6], the opacity of films can vary depending on the content of amylose, whose molecules of linear nature tend to favor tight hydrogen bonds between the hydroxyl groups of adjacent chains. Therefore, the BFS incorporated with banana peel extract had an effect on the optical properties of the resulting film.

#### 2.1.8. Antimicrobial Activity of the Films

**Figure 2:**
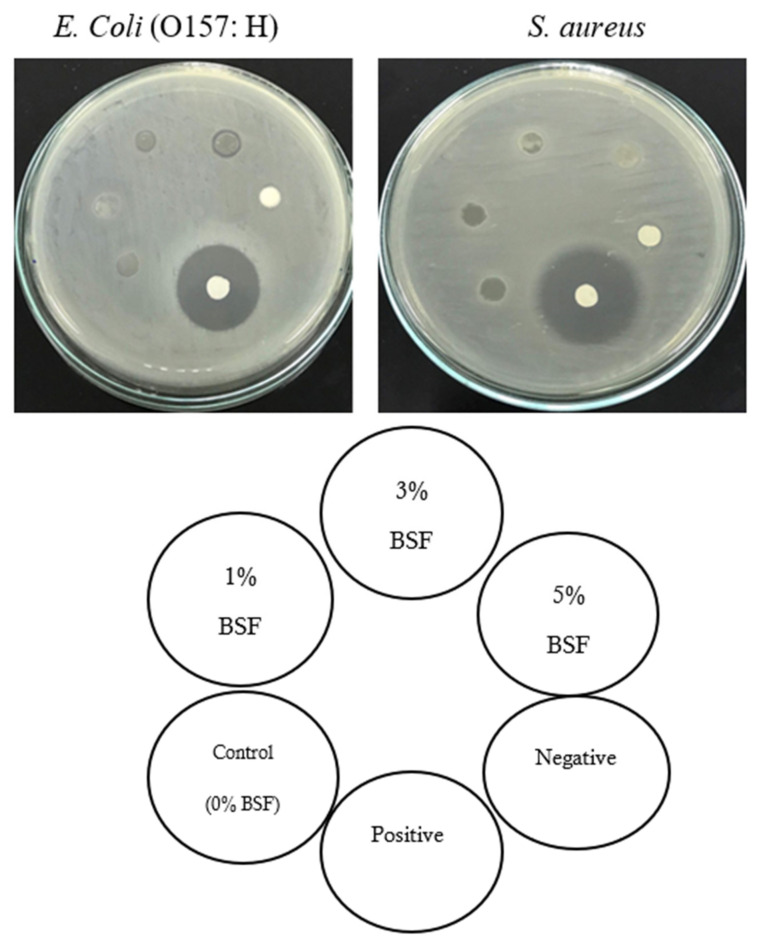
Antimicrobial properties of banana starch films incorporated with banana peel extract at different concentrations against *E. coli* (O157: H7) and *S. aureus* (TISTR 1466). Negative: distilled water (30 μg/disc); Positive: ampicillin (30 μg/disc).

The antimicrobial activity of the BSF incorporated with banana peel extract at different concentrations is shown in Figure 2. The control film and the BSF at a concentration of 1 (%, *w/v*) banana peel extract did not show any inhibitory activity against Gram-negative bacteria (*E. coli* O157: H7) and Gram-positive (*S. aureus* TISTR 1466) food borne pathogenic bacteria. On the other hand, the BSF at the concentration of 3 and 5 (%, *w/v*) banana peel extract presented inhibition activity only against *E. coli* (O157: H7), and the value of the inhibition zone area ranged between 0.1 and 0.2 mm, respectively. As expected, the antimicrobial activity of the BSF was mainly attributed to phenolic compounds of banana peel extract. In the present study, the inhibitory zone presented much lower values due to the lower banana peel extract concentration that was applied to the films.

### 2.2. Quality Attributes of Minced Pork Wrapped with Developed Banana Starch Film

#### 2.2.1. Color Changes of Minced Pork during Storage

Color is one of the most important attributes of meat quality. The L*, a*, b*, ΔE and the visual quality of minced pork wrapped with the BSF and PVC films during refrigeration (4 ± 1 °C) for 7 days are shown in Table 4. All minced pork showed an increase in L* value when storage was extended (*p* < 0.05). However, the samples wrapped with the BSF showed significantly different lower L* than the PVC film during refrigeration (*p* < 0.05), while the a* value of the samples wrapped with both films exhibited a significantly different decrease during storage (*p* < 0.05). This indicated that the sample became less red in color. The redness of minced pork depends on the myoglobin content in the meat. The increase in b* value was probably related to an increase in the thiobarbituric acid reactive substances (TBARS). Glycation occurs in vivo through the covalent binding of aldehyde or ketone groups of reducing sugars to free the amino groups of proteins, forming advanced glycation end product structures that may have a yellowish-brown color or fluorescence [28]. The minced pork also showed high ΔE values in both films, whereas the PVC film retained the external color of minced pork better during refrigerated storage. Hence, myoglobin oxidation was postulated to occur, which explains the reduction in the redness. This could be due to the antioxidant properties of the BSF. This suggests that the sustained release of banana peel extract from the film to the flesh surface resulted in retarded oxidation. In addition, the BSF wrapped sample could protect against the loss of redness in the minced pork.

#### 2.2.2. Weight Loss, TBARS and pH Changes of Minced Pork during Storage

**Figure 3:**
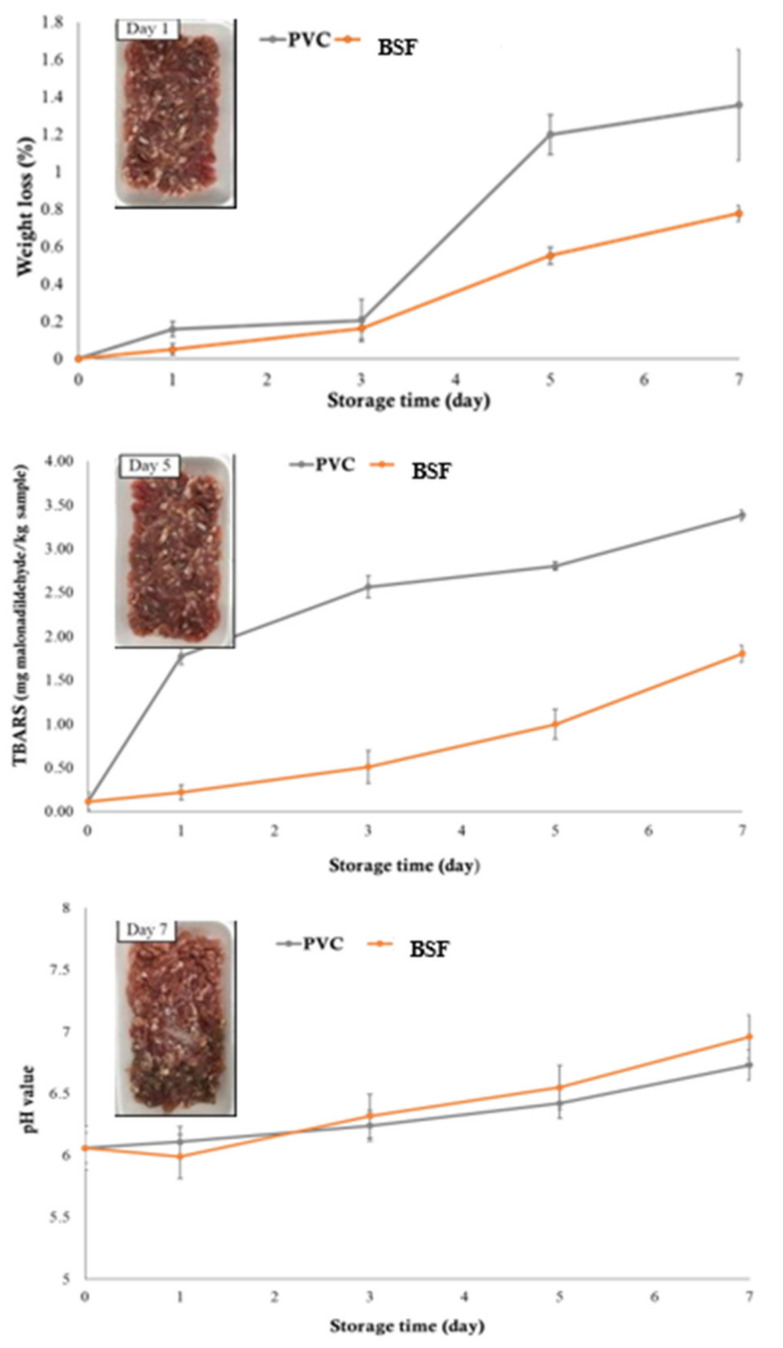
Weight loss, thiobarbituric acid reactive substances (TBARS), and pH changes of the wrapped minced pork with PVC and BSF during refrigerated storage for 7 days.

The weight loss of minced pork wrapped with the BSF (5% incorporation) was less than that wrapped with PVC film (Figure 3). These values had a significantly different increase when the storage time increased (*p* < 0.05). The weight loss value of the samples wrapped with the BSF was 0.78%, while the sample wrapped with PVC was 1.36% after 7 days of storage. In addition, the higher weight loss of the sample wrapped with PVC may be caused by the prevention mechanism of external moisture to enter into packed meat. While the minced pork that was wrapped with the BSF had a higher weight gain during storage because of its hydrophilic nature, banana starch absorbed moisture from the atmosphere. Therefore, the BSF could hold moisture inside the minced pork. 

The TBARS of minced pork wrapped with the BSF incorporating 5 (%, *w/v*) banana peel extract and PVC film was significantly increased during refrigerated storage conditions (*p* < 0.05) (Figure 3). The minced pork wrapped with the BSF showed lower TBARS than the minced pork wrapped with PVC film (*p* < 0.05). This suggests that lipid oxidation in minced pork could be delayed when the BSF was applied. However, the BSF could not completely inhibit the oxidation reaction. This is probably due to the low migration rate of active compounds (banana peel extract) incorporated in the BSF. Additionally, the oxidation retardation is probably because UV light was inhibited from penetrating the wrapped film into the samples. Benjakul et.al. [29] reported that lipid oxidation in meat can be initiated and increased by photosensitized oxidation, autoxidation, lipoxygenase, and peroxidase. In lipid oxidation, free radicals bind with hydrogen to form hydro peroxide. The continuous increase in free radicals of fatty acid, which react with oxygen to form hydro in TBARS values was observed in minced pork wrapped with developed film during 7 days of storage (*p* < 0.05). Therefore, the BSF incorporated with banana peel extracts enhanced the antioxidant properties that resulted in a lower TBARS range from 1.79 mg malonaldehyde/kg sample than that wrapped with PVC film (3.38 mg malonaldehyde/kg sample). The delay of oxidation is probably due to low UV light passing through the film. Additionally, banana peel extract has been reported to be a good source of antioxidants [15,17].

The pH values of minced pork wrapped with the BSF incorporating 5 (%, *w/v*) banana peel extract and PVC film during refrigerated storage for 7 days are shown in Figure 3. The pHs of all minced pork were significantly different (*p* < 0.05). The pH of the minced pork wrapped with the BSF slightly increased from 5.99 to 6.96 after storage for 7 days. When compared with the sample wrapped with PVC film, the BSF showed a higher pH than that of PVC film. The increase in pH value for all of the minced pork samples wrapped with the BSF was observed when compared with the sample wrapped with PVC film. This is related to the higher microbial count in the wrapped samples using the BSF.

#### 2.2.3. Microbiological Quality of Minced Pork during Storage

The total plate count, yeasts and molds of minced pork wrapped with the BSF incorporating 5 (%, *w/v*) banana peel extract and PVC film under refrigerated storage for 7 days are shown in Table 5. The sample wrapped with the BSF showed higher total plate count, yeasts, and molds than the sample wrapped with PVC film (*p* < 0.05). Meat and meat products provide excellent growth media for a variety of microflora (bacteria, yeasts and molds), some of which are pathogens [30]. The mentioned microbial groups are considered to be food spoilage microorganisms, and their presence in high amounts could affect the organoleptic properties of the samples. 

The relatively high amount of yeasts and molds may also cause the formation of slime and greening on the surface of the sample [18,19]. The total plate count of the sample wrapped with the BSF was 4.14 log CFU/g after 7 days of storage, which was higher than the PVC film. For yeasts and molds of the sample wrapped with the BSF, they were also higher than the PVC film. Starch is the primary component of unripe banana and it undergoes several changes during ripening [4,31]. Foodborne microorganisms can derive energy from carbohydrates, alcohols, and amino acids. Most microorganisms will metabolize simple sugars such as glucose. Others can metabolize more complex carbohydrates, such as starch or cellulose found in plant foods, or glycogen found in muscle foods.

## 3. Materials and Methods

### 3.1. Chemicals and Reagents

Trichloroacetic acid (TCA), thiobarbituric acid (TBA), 1,1,3,3-tetramethoxypropane, and glycerol were obtained from Merck (Frankfurter Straße, Darmstadt, Germany). Mueller-hinton broth was purchased from Difco (Lawrence, KS, USA). Trolox ((±)-6-Hydroxy-2,5,7,8-tetramethylchromane-2-carboxylic acid) and DPPH (2,2-diphenyl-1-picryhydrazyl) were purchased from Aldrich (Riedstraße, Steinheim am Albuch, Germany). Plate Count Agar and Potato Dextrose Agar were obtained from the Biological Laboratories, Mae Fah Luang University. All other chemicals and solvents in this study were analytical grade.

### 3.2. Sample Preparation

#### 3.2.1. Banana Flour and Peel Powder Preparation

Unripe banana (*Cavendish* spp.) was obtained from the Phaya Mengrai Kankaset in Chiang Rai, Thailand. Banana flour was prepared according to the method of Alves et al. [32] with some modification. Unripe banana fruits and peel were dipped in 50 ppm of chlorine solution for 15 min, cut into 5-mm slices and immediately dipped in a 0.1% citric acid solution for 2 h. Unripe banana fruit slices were dried at 60°C for 16 h, while banana peel slices were dried at 55 °C for 16–18 h in a tray dryer. The dried slices were ground in a pulverizing machine (RT-08, Rong Tsong Precision Technology Co., Taiching, Taiwan) and passed through a 60-mesh sieve. Then, banana flour and peel were stored at −20 °C in a vacuum plastic bag before further use.

#### 3.2.2. Banana Starch Preparation

Banana starch was prepared using a water-alkaline extraction process described by Salama, Z. H. [33] with some modification. Banana flour (100 g) was added to 1 L distilled water and macerated at low speed for 20 min; the homogenate was sieved through 60-mesh screens. The collected milk was centrifuged at 4000 g for 10 min, and 1 L NaOH solution 0.2 (%, *w/v*) was added to the sediment to remove soluble fiber. The cleaned starch sediment was dispersed in distilled water and repeatedly washed until neutrality. The resulting materials were then placed on trays and dried in a vacuum oven at 45 °C for 24 h. The dried banana starch was ground and sieved through a 120-mesh sieve.

#### 3.2.3. Banana Peel Extraction

Extraction of banana peel was performed according to the method of Aboul-Enein et al. [33] with some modification. One hundred grams of dried banana peel was dispensed in 1 L of 80% acetone, shaking at room temperature for 24 h. The mixture was filtered through Whatman No 1 filter paper and the extraction step was repeated twice. The filtrate was then concentrated at 60 °C in a rotary evaporator before being freeze dried for 15–16 h. The peel extract was ready to use and freshly prepared every time. 

#### 3.2.4. Preparation of Banana Starch Film

Banana starch solution was prepared according to the method of Pelissari et al. [6]. Banana starch was dissolved at a concentration of 4 (%, *w/v*) with distilled water and then stirred at 81 °C for 15 min. Glycerol at a concentration of 25 (%, *w/v*) was used as a plasticizer mix on stirrer under gentle stirring and then the solution was cooled. The film forming solution was prepared by mixing different concentrations, 0, 1, 3, and 5 (%, *w/v*), of banana peel extract with banana starch, and then four grams of solution was cast on rimmed silicone resin plates (18 cm × 21 cm) and dried at 54 °C. The films were conditioned in a desiccator under 50% Relative Humidity, at 25 °C for 48 h. The final dried films were manually peeled.

### 3.3. Banana Starch Film Characterization

#### 3.3.1. Thickness

The film thickness was measured by using a hand-held micrometer (Bial Pipe Gauge, Peacock Co., Tokyo, Japan). Nine random locations around each of the ten film samples were used for thickness determination.

#### 3.3.2. Color

The color of the film was determined by using a Color Quest XE (Hunter Lab, Virginia) and expressed as the average L* value (lightness), a* value (redness/greenness) and b* value (yellowness/blueness) of the film. Measurement was performed in triplicate.

#### 3.3.3. Light Transmission and Transparency

The light transmission of the films against ultraviolet (UV) and visible light was measured according to the method of Kaewprachu et al. [23] at select wavelengths between 200 and 800 nm using a UV-Vis spectrophotometer (G105 UV-VIS, Thermo Scientific Inc., MA, USA). The transparency value of the film was calculated according to the equation (Han and Floros 1997):

Transparency = −log T600/x(1)

where T600 is the fractional transmittance at 600 nm, and x is the film thickness (mm).

#### 3.3.4. Mechanical Properties

Prior to testing the mechanical properties, the films were conditioned for 48 h at 50 ± 5% Ralative Humidity (RH) at 25 °C. The tensile strength (TS) and elongation at break (EAB) were determined according to the method of American Society for Testing and Materials (ASTM) by using a Universal Testing Machine (Lloyd Instrument, Hampshire, UK). Three samples (2 × 5 cm) with an initial grip length of 3 cm were used for testing. The cross-head speed was set at 30 mm/min with 100 N load cell use. 

#### 3.3.5. Differential Scanning Calorimetry 

The thermal properties of film were analyzed according to the method of Pe-lissari et al. [6] on a differential scanning calorimeter (DSC; TA-Instruments, model 2920, PA, USA) equipped with a cooling system. The film (7–8 mg) was accurately weighed into aluminum pans, hermetically sealed, and scanned over the range of −60 to 150 °C with a heating rate of 10 °C/min in a nitrogen atmosphere (20 mL/min). The empty aluminum pan was used as a reference.

#### 3.3.6. Moisture Content

The moisture content of films was analyzed according to the method of Rhim and Wang [34]. Film samples were cut into squares of 3 cm × 3 cm and were first weighed (W1). The film samples were placed in an oven at 105 °C, and after drying for 24 h, the films were weighed (W2) again. The water content (WC) was determined as the percentage of initial film weight lost during drying and reported on a wet basis.

WC (%) = 100(W1 − W2)/W1(2)

Triplicate measurements of WC were conducted for each type of film, and an average was taken as the result.

#### 3.3.7. Water Vapor Permeability 

The films’ WVP was measured by using a modified ASTM method, as described by Kaewprachu et al. [23]. The films were sealed onto a permeation cup containing silica gel (0% RH) with silicone vacuum grease and an O-ring to hold the film in place. The cups were then placed in a desiccator saturated with water vapor at 25 °C. The cups were weighed at 1 h intervals over a period of 8 h, and the films’ WVP was calculated as follows:

WVP = WXA-1t-1 (P2 − P1)-1(3)

W is the weight gain of the cup (g); X is the film thickness (mm); A is the area of exposed film (cm^2^); t is the time of gain (h); and (P2 − P1)^−1^ is the vapor pressure differences across the film (Pa). The WVP was expressed as g mmh^−1^ cm^−2^ Pa^−1^.

#### 3.3.8. Film Solubility

The film solubility was determined according to the method of Kaewprachu et al. [15]. The weighed conditioned films were placed in 10 mL of distilled water in a 50 mL centrifuge tube, then shaken at a speed of 250 rpm for 24 h. The un-dissolved debris was removed by centrifugation at 3000 g for 20 min. The pellet was dried at 105 °C for 24 h and weighed. The weight of the solubilized dry matter was calculated by subtracting its difference from the initial weight of the dry matter using the following equation:

Film solubility (%) = {(W0 − Wf)/W0} × 100(4)

W0 was the initial weight of the film, and Wf was the weight of the un-dissolved film residue. 

#### 3.3.9. Antioxidant Properties

Film extract solution was prepared according to the method described in Tongnuanchan et al. [35]. The film (25 mg) was added with 3 mL of distilled water. After stirring for 3 h, the mixtures were centrifuged at 3000 g for 10 min and the supernatant obtained was then determined for DPPH radical scavenging activity. The film extract solution (1.5 mL) was added to 0.15 mM 2,2-diphenyl-1-picryl hydrazyl (DPPH) in 95% ethanol (1.5 mL), followed by mixing, and then kept in the dark for 30 min at room temperature. The DPPH assay solution recorded the absorbance at 517 nm using a spectrophotometer. The films’ antioxidant activity was expressed as the percentage of DPPH radical scavenging activity.

#### 3.3.10. Antimicrobial Activity

Antimicrobial activity of the films was performed using the agar diffusion method according to the method of Kaewprachu et.al. [23]. *E. coli* (O157: H7) and *S. aureus* (TISTR 1466) were cultured into Muller-Hinton broth (MHB) and incubated in a shaker incubator at 37 °C for 18–24 h. A loopful of the microorganism working stocks was streaked onto a Muller-Hinton (MH) agar plate and further incubated at 37 °C for 18–24 h to obtain a single colony. The optical density of the cultures was adjusted to 0.5 McFarland turbidity standards with 0.85% normal saline and then inoculated on MH agar plates using a sterile swab. A film sample was cut into a circular shape (6 mm in diameter) and then placed on a Muller-Hinton (MH) agar surface, which had been inoculated with microorganisms. Ampicillin (30 μg/disc) was used in this study as antibiotics for the strains tested. After incubation (37 °C for 18–24 h), the plate was investigated for inhibition zones on the film discs.

### 3.4. Application of the Banana Starch Films

Minced pork was purchased from the Tesco Lotus supermarket in Chiang Rai, Thailand. Minced pork samples (50 g each) were placed on a polystyrene (PS) tray (9.5 cm × 15.9 cm × 2 cm). The banana starch film incorporated with banana peel extract and PVC films were used to cover the minced pork in the PS tray. This was performed in atmosphere conditions and by avoiding contact between the film and the minced pork [15]. All prepared samples were stored in a refrigerator (4 ± 1 °C) and monitored at the days 0, 1, 3, 5, and 7 for quality attributes. 

### 3.5. Determinations of Quality of Minced Pork

#### 3.5.1. Color

The color of the minced pork was determined according to the method of Kaewprachu et al. [15] by using a Color Quest XE (Hunter Lab, Virginia) and expressed as the average L* value (lightness), a* value (redness/greenness) and b* value (yellow-ness/blueness) of the film. Measurement was performed in triplicate and then the ∆E value was calculated.

∆E = √ ([(L − L*)2 + (a − a*)2 + (b − b*)2])(5)

where this denotes values at 0 day of storage time, and L*, a*, and b* denote values at days 1, 3, 5, and 7 of the storage. 

#### 3.5.2. Weight Loss 

The weight loss of the minced pork was determined according to the method of Herring et al. [36] by comparing the weights of the initial sample with the weight of the sample after each day of storage:

Weight loss (%) = {(W0 − Wf)/W0} × 100(6)

W0 was the initial weight of the samples and Wf was the weight of the sample after each day of storage. 

#### 3.5.3. PH Determination

The pH of the minced pork was determined according to the method of Kaewprachu et.al. [18]. Ten grams of the minced pork was homogenized with 50 mL of chilled distilled water before being subjected to pH measurement by using a digital pH meter (Model pH 510, Eutech Instrument, Ayer Rajah Crescent, Singapore).

#### 3.5.4. Lipid Oxidation

Thiobarbituric acid reactive substances (TBARS) were determined as described by Buege and Aust [37]. One gram of minced pork sample was homogenized with 5 mL of solution containing 0.0375 g/100 g TBA, 15 g/100 g TCA, and 0.25M HCl. The mixture was heated at 95 °C for 10 min. The heated sample was cooled and centrifuged at 3600 g for 20 min. The absorbance of the supernatant was measured at 532 nm. As a standard curve, 1, 1, 3, 3-tetramethoxypropane at a concentration ranging from 0 to 10 µg/mL were used. TBARS was expressed as the mg malonaldehyde/kg sample. 

#### 3.5.5. Microbiological Quality

Microbial analysis was determined according to the method described in Siripatrawan and Noipha [38]. Minced pork (25 g) was homogenized in 225 mL of 1 g/100 g peptone and 0.5% (*w/v*) NaCl for 2 min using a Stomacher Lab Blender (Seward, Model 400 CTF, Bangkok, Thaliand). The prepared samples were spread-plated on Plate Count Agar and incubated at 37 °C for 24 h for total plate counts. For yeasts and molds, the prepared samples were pour-plated on Potato Dextrose Agar and incubated at 25 °C for 5 days. For lactic acid bacteria, the prepared samples were pour-plated on Lactobacillus MRS Agar and incubated at 37 °C for 72 h.

### 3.6. Statistical Analysis

Analysis of variance (ANOVA) was performed. The mean comparison was carried out by Duncan’s Multiple Range Test. Significance of difference was defined at *p* < 0.05. The analysis was performed by using an SPSS package (SPSS 16.0 for window, SPSS Inc., Chicago, IL, USA).

## 4. Conclusions

The present study was focused on the development and application of banana starch film incorporated with banana peel extract in order to improve the quality attributes and extend the shelf life of minced pork. The BSF presented low mechanical properties and water vapor permeability as compared with PVC film. For barrier properties to UV light, the BSF had better barrier properties than PVC film. Furthermore, the application of the BSF could maintain some quality attributes of minced pork, especially lipid oxidation. However, the BSF was not effective for the inhibition of microbial growth during refrigerated storage. Moreover, the results showed that the BSF may be used as active packaging for any food products as it showed antioxidant (dominant) and antimicrobial properties. 

## Figures and Tables

**Table 1:** Thickness, mechanical properties, and thermal properties of banana starch films incorporated with banana peel extract at different concentrations.

BSF with Banana Peel Extract	Thickness	Tensile Strength (TS)	Elongation at Break (EAB)	Thermal Properties	
(%, **w*/*v**)	(mm)	(MPa)	(%)	Tm (°C)	ΔH (J/g)
Control (0)	0.030 ± 0.004 ^b^	31.20 ± 0.54 ^b^	15.63 ± 2.66 ^b^	95.50 ± 2.89 ^b^	273.90 ± 8.32 ^a^
1	0.048 ± 0.004 ^a^	4.43 ± 0.69 ^d^	9.66 ± 2.09 ^b^	89.83 ± 0.00 ^c^	219.70 ± 3.53 ^c^
3	0.048 ± 0.004 ^a^	5.28 ± 0.22 ^c^	11.34 ± 0.49 ^b^	97.61 ± 0.10 ^ab^	263.60 ± 3.17 ^b^
5	0.047 ± 0.003 ^a^	4.48 ± 0.11 ^d^	12.16 ± 0.93 ^b^	98.50 ± 0.00 ^a^	192.30 ± 4.83 ^d^
PVC	0.010 ± 0.00 ^c^	44.10 ± 0.28 ^a^	238.75 ± 22.30 ^a^	ND *	ND

Different superscripts (a, b, c, d, ab) in each column are significantly different (*p* < 0.05). BSF: banana starch film; PVC: polyvinyl chloride; Tm: melting temperature; ΔH: enthalpy; ND; not determine. Values are given as mean ± SD from triplicate determinations.

**Table 2:** Moisture content, film solubility, water vapor permeability, and DPPH scavenging activity of banana starch films incorporated with banana peel extract at different concentrations.

BSF with Banana Peel Extract (%, *w/v*)	Moisture Content (%)	Film Solubility (%)	WVP (× 10^−10^g mm/sm^2^ Pa)	DPPH Scavenging Activity (%)
Control (0)	26.3 ± 3.24 ^b^	26.3 ± 1.68 ^c^	3.74 ± 0.97 ^b^	66.40 ± 1.04 ^c^
1	27.7 ± 2.97 ^b^	33.2 ± 3.97 ^b^	9.56 ± 0.47 ^a^	67.30 ± 0.98 ^c^
3	29.1 ± 1.41 ^ab^	38.9 ± 2.50 ^ab^	9.96 ± 0.74 ^a^	77.00 ± 1.63 ^b^
5	33.7 ± 2.65 ^a^	40.8 ± 4.75 ^a^	11.0 ± 1.62 ^a^	97.90 ± 5.42 ^a^
PVC	ND *	ND	1.15 ± 0.09 ^c^	ND

Different superscripts (a, b, c, d, ab) in each column are significantly different (*p* < 0.05). Values are given as mean ± SD from triplicate determinations. WVP: water vapor permeability; ND *: not detected.

**Table 3:** Light transmission and transparency of banana starch films incorporated with banana peel extract at different concentrations in comparison with PVC film.

BSF with Banana Peel Extract (%, *w/v*)	Transmittance (%T) at Wavelength (nm)								Transparency ^*^
	200	280	350	400	500	600	700	800	
Control (0)	27.1	46.9	48.8	49.1	49.3	49.6	50.4	51.7	3.23 ± 0.02 ^b^
1	24.3	40.5	41.3	42.6	42.8	43.1	43.4	44.5	2.97 ± 0.04 ^c^
3	21.6	31.5	32.4	32.6	33.6	33.7	33.8	35.0	2.85 ± 0.01 ^d^
5	18.9	30.5	31.7	31.9	32.5	33.0	33.6	34.2	2.85 ± 0.02 ^d^
PVC	12.1	84.8	87.9	89.5	90.3	91.3	91.6	91.9	3.95 ± 0.00 ^a^

Different superscripts (a, b, c, d) in each column are significantly different (*p* < 0.05). * Values are given as mean ± SD from triplicate determinations.

**Table 4:** Color attributes of wrapped minced pork during refrigeration (4 ± 1 °C) for 7 days.

Storage Time (Days)	Film Wrap	L^*^	a^*^	b^*^	ΔE
0	BSF	42.8 ± 0.62 ^g^	5.42 ± 0.26 ^a^	7.03 ± 0.63 ^f^	-
	PVC	42.8 ± 0.62 ^g^	5.42 ± 2.56 ^a^	7.03 ± 0.63 ^f^	-
1	BSF	47.5 ± 1.35 ^f^	4.42 ± 0.11 ^bc^	7.96 ± 0.33 ^ef^	5.79 ± 2.35 ^e^
	PVC	50.9 ± 0.50 ^e^	4.85 ± 0.60 ^ab^	8.30 ± 0.41 ^ef^	9.14 ± 1.22 ^d^
3	BSF	51.2 ± 1.10 ^e^	3.88 ± 0.33 ^cd^	9.83 ± 0.91 ^cd^	9.11 ± 1.66 ^d^
	PVC	54.4 ± 1.23 ^cd^	4.26 ± 0.39 ^c^	8.89 ± 0.47 ^de^	11.8 ± 1.72 ^cd^
5	BSF	53.5 ± 1.43 ^d^	3.50 ± 0.06 ^de^	10.7 ± 0.83 ^bc^	11.5 ± 1.59 ^cd^
	PVC	56.3 ± 1.40 ^b^	3.85 ± 0.25 ^cd^	11.4 ± 1.30 ^b^	14.4 ± 1.35 ^b^
7	BSF	55.6 ± 0.73 ^bc^	2.97 ± 0.11 ^e^	11.7 ± 0.88 ^ab^	13.9 ± 0.93 ^bc^
	PVC	60.6 ± 0.50 ^a^	3.48 ± 0.44 ^de^	12.9 ± 0.38 ^a^	18.8 ± 0.60 ^a^

Values (*n* = 3) are given as mean ± SD. Different letters (c, d, cd, e, f, g, bc, etc) indicate significant difference (*p* < 0.05). L*: lightness; a*: redness/greenness; b*: yellowness/blueness.

**Table 5:** The microbiological quality of minced pork wrapped with banana starch film incorporated with banana peel extract and PVC films (log CFU/g).

Storage Time (Days)	Film	Total Plate Count (log CFU/g)	Yeast and Molds (log CFU/g)
0	BSF	2.68 ± 0.03 ^a^	2.27 ± 0.09 ^a^
	PVC	2.68 ± 0.23 ^a^	2.27 ± 0.09 ^a^
1	BSF	3.72 ± 0.04 ^b^	2.55 ± 0.18 ^a^
	PVC	2.99 ± 0.03 ^a^	2.36 ± 0.11 ^a^
3	BSF	3.89 ± 0.03 ^a^	2.79 ± 0.08 ^a^
	PVC	3.72 ± 0.04 ^b^	2.55 ± 0.09 ^b^
5	BSF	4.04 ± 0.02 ^a^	2.82 ± 0.11 ^a^
	PVC	3.83 ± 0.06 ^b^	2.63 ± 0.16 ^a^
7	BSF	4.14 ± 0.02 ^a^	2.90 ± 0.02 ^a^
	PVC	4.07 ± 0.01 ^b^	2.79 ± 0.09 ^a^

Data represent mean ± SD of triplicate determinations. CFU: colony forming unit. Different superscripts in the same column indicate significant differences (*p* < 0.05).

## Data Availability

Data sharing not applicable.

## Sample Availability

Not available.

## References

[1] Leceta I., Guerrero P., De la Caba K. (2013). Functional properties of chitosan-based films. Carbohydr. Polym..

[2] Sartori T., Menegalli F.C. (2016). Development and characterization of unripe banana starch films incorporated with solid lipid microparticles containing ascorbic acid. Food Hydrocolloids.

[3] Cazón P., Velazquez G., Ramírez J.A., Vázquez M. (2017). Polysaccharide-based films and coatings for food packaging: A re-view. Food Hydrocolloids.

[4] Restrepo A.E., Rojas J.D., Garcia O.R., Sanchez L.T., Pinzon M.I., Villa C.C. (2018). Mechanical, barrier, and color properties of banana starch edible films incorporated with nanoemulsions of lemongrass (Cymbopogon citratus) and rosemary (Ros-marinus officinalis) essential oils. Food Sci. Technol. Int..

[5] Ramírez-Hernández A., Aparicio-Saguilán A., Reynoso-Meza G., Carrillo-Ahumada J. (2017). Multi-objective optimization of process conditions in the manufacturing of banana (*Musa paradisiaca* L.) starch/natural rubber films. Carbohydr. Polym..

[6] Pelissari F.M., Andrade-Mahecha M.M., Sobral P.J.d.A., Menegalli F.C. (2012). Isolation and characterization of the flour and starch of plantain bananas (*Musa paradisiaca*). Starch-Stärke.

[7] Tibolla H., Pelissari F., Martins J., Lanzoni E., Vicente A., Menegalli F., Cunha R. (2019). Banana starch nanocomposite with cellulose nanofibers isolated from banana peel by enzymatic treatment: In vitro cytotoxicity assessment. Carbohydr. Polym..

[8] Nieto-Suaza L., Acevedo-Guevara L., Sánchez L.T., Pinzón M.I., Villa C.C. (2019). Characterization of Aloe vera-banana starch composite films reinforced with curcumin-loaded starch nanoparticles. Food Struct..

[9] Mohapatra D., Mishra S., Sutar N. (2010). Banana and its by-product utilisation: An overview. J. Sci. Ind. Res..

[10] Borges C., Maraschin M., Coelho D., Leonel M., Gomez H., Belin M., Diamante M., Amorim E., Gianeti T., Castro G. (2020). Nutritional value and antioxidant compounds during the ripening and after domestic cooking of bananas and plantains. Food Res. Int..

[11] Orsuwan A., Sothornvit R. (2017). Effect of banana and plasticizer types on mechanical, water barrier, and heat sealability of plasticized banana-based films. J. Food Process. Preserv..

[12] Kaur L., Dhull S.B., Kumar P., Singh A. (2020). Banana starch: Properties, description, and modified variations - A review. Int. J. Biol. Macromol..

[13] Nur Hanani Z.A., Abdullah S. (2016). Development of green banana (*Musa paradisiaca*) as potential food packaging films and coatings. Int. J. Adv. Sci..

[14] Daudt R., Avena-Bustillos R., Williams T., Wood D., Külkamp-Guerreiro I., Marczak L., McHugh T. (2016). Comparative study on properties of edible films based on pinhão (*Araucaria angustifolia*) starch and flour. Food Hydrocoll..

[15] Chabuck Z.A.G., Al-Charrakh A.H., Hindi N.K.K., Hindi S.K.K. (2013). Antimicrobial effect of aqueous banana peel extract, Iraq. Res. Gate. Pharm. Sci..

[16] Kapadia S.P., Pudakalkatti P.S., Shivanaikar S. (2015). Detection of antimicrobial activity of banana peel (*Musa paradisiaca* L.) on Porphyromonas gingivalis and Aggregatibacter actinomycetemcomitans: An in vitro study. Contemp. Clin. Dent..

[17] Zhang W., Li X., Jiang W. (2020). Development of antioxidant chitosan film with banana peels extract and its application as coating in maintaining the storage quality of apple. Int. J. Biol. Macromol..

[18] Kaewprachu P., Osako K., Benjakul S., Rawdkuen S. (2015). Quality attributes of minced pork wrapped with catechin–lysozyme incorporated gelatin film. Food Packag. Shelf Life.

[19] Pattarasiriroj K., Kaewprachu P., Rawdkuen S. (2020). Properties of rice flour-gelatine-nanoclay film with catechin-lysozyme and its use for pork belly wrapping. Food Hydrocoll..

[20] Gutiérrez T.J., Morales N.J., Tapia M.S., Pérez E., Famá L. (2015). Corn starch 80:20 “waxy”: Regular, “native” and phos-phated, as bio-matrixes for edible films. Procedia Mater. Sci..

[21] Pérez E., Segovia X., Tapia M.S., Schroeder M. (2012). Native and cross-linked modified Dioscorea trifida (cush-cush yam) starches as bio-matrices for edible films. J. Cell. Plast..

[22] Gutiérrez T.J., Álvarez K. (2016). Physico-chemical properties and in vitro digestibility of edible films made from plantain flour with added Aloe vera gel. J. Funct. Foods.

[23] Kaewprachu P., Rungraeng N., Osako K., Rawdkuen S. (2017). Properties of fish myofibrillar protein film incorporated with catechin-Kradon extract. Food Packag. Shelf Life.

[24] Gutiérrez T.J., Suniaga J., Monsalve A., García N.L. (2016). Influence of beet flour on the relationship surface-properties of edible and intelligent films made from native and modified plantain flour. Food Hydrocoll..

[25] Verbeek C.J.R., Bier J.M., Sharma S.K., Mudhoo A. (2011). Synthesis and characterization of thermoplastic agro-polymers. Handbook of Applied Biopolymer Technology.

[26] Ma X., Jian R., Chang P.R., Yu J. (2008). Fabrication and characterization of citric ccid-modified ctarch nanoparti-cles/plasticized-xtarch composites. Biomacromolecules.

[27] Orsuwan A., Shankar S., Wang L.-F., Sothornvit R., Rhim J.-W. (2016). Preparation of antimicrobial agar/banana powder blend films reinforced with silver nanoparticles. Food Hydrocoll..

[28] Wu C.-H., Huang S.-M., Lin J.-A., Yen G.-C. (2011). Inhibition of advanced glycation endproduct formation by foodstuffs. Food Funct..

[29] Benjakul S., Visessanguan W., Phongkanpai V., Tanaka M. (2005). Antioxidative activity of caramelisation products and their preventive effect on lipid oxidation in fish mince. Food Chem..

[30] Dave D., Ghaly A.E. (2011). Meat Spoilage Mechanisms and Preservation Techniques: A Critical Review. Am. J. Agric. Biol. Sci..

[31] Zhang P., Whistler R.L., Bemiller J.N., Hamaker B.R. (2005). Banana starch: Production, physicochemical properties, and digestibility - A review. Carbohydr. Polym..

[32] Alves L.A.A.D.S., Lorenzo J.M., Gonçalves C.A.A., Dos Santos B.A., Heck R.T., Cichoski A.J., Campagnol P.C.B. (2016). Production of healthier bologna type sausages using pork skin and green banana flour as a fat replacers. Meat Sci..

[33] Salama Z.H. (2016). Identification of phenolic compounds from banana peel (*Musa paradaisica* L.) as antioxidant and antimicrobial agents. J. Chem. Pharm. Res..

[34] Rhim J.-W., Wang L.-F. (2013). Mechanical and water barrier properties of agar/κ-carrageenan/konjac glucomannan ternary blend biohydrogel films. Carbohydr. Polym..

[35] Tongnuanchan P., Benjakul S., Prodpran T. (2012). Properties and antioxidant activity of fish skin gelatin film incorporated with citrus essential oils. Food Chem..

[36] Herring J.L., Jonnalongadda S.C., Narayanan V.C., Coleman S.M. (2010). Oxidative stability of gelatin coated pork at refrigerated storage. Meat Sci..

[37] Buege J.A., Aust S.D. (1978). Microsomal lipid peroxidation. Methods Enzymol..

[38] Siripatrawan U., Noipha S. (2012). Active film from chitosan incorporating green tea extract for shelf life extension of pork sausages. Food Hydrocoll..

